# Research on Digital News Distribution Based on Cognitive Neuroscience

**DOI:** 10.1515/tnsci-2019-0009

**Published:** 2019-04-23

**Authors:** Xiaomei Zheng

**Affiliations:** 1 School of Literature and Journalism, Chongqing Technology and Business University, Chongqing 400067, China

**Keywords:** Cognitive neuroscience, Cognitive and neural transmission, Digital News Distribution

## Abstract

Updated computer and brain science have profoundly changed journalism and communication as well as the media industry. The application of related technologies and theories represented by big data technology, cognitive neuroscience and intelligent technology may bring about a paradigmatic shift in journalism and communication. On the basis of combing the frontier development of cognitive neuroscience at home and abroad, this paper introduces the cases of applying the cognitive neurological method in communication studies and studies the application situation of the communicating effect in the field of advertising, photos, news webs, and films and television. This paper also discusses the important significance of relevant frontier results for the development of journalism and communication discipline.

## 1 Introduction

The rapid development of Internet communication, intelligent mobile communication, big data, artificial intelligence and other technologies not only bring new challenges and opportunities for the traditional media industry, but also for the humanities and social sciences, including communication science[1, 2, 3, 4]. The new cross-disciplinary technological wave has spawned computational communication which is based on mature data and calculation methods. Meanwhile, it also starts a new page of exploring communication behaviours and relations from the perspective of brain science.

The human brain is the physiological basis of human thoughts, emotions, imaginations and behaviours and its operation law is a prerequisite for human beings to understand themselves. Due to the lack of reliable experimental methods, the study methods for human cognition in traditional humanities and social sciences have always been limited to subjective observation, introspection and textual analysis, questionnaire and interview based on observation and introspection, so the research process and conclusion are inevitably affected by subjective factors [5, 6, 7]. Moreover, most of these studies focus on the conscious level. Due to the limitation of research methods, the study of subconscious factors is relatively weak [8]. However, precisely the subconscious factors have a significant impact on people’s emotions, thoughts and behaviours. With the continuous enrichment of relevant results, it is expected to form a new paradigm for the research on computational communication.

## 2 Discovery of the Unconscious Dimension of Communication

Ohm’s long-term research on the subconscious role of advertising communication, his research methods are the reference to the theory and research framework of cognitive neuroscience. The brain is the centre of human thoughts, feelings and behaviours. The realization of communication effects is inseparable from the related neural activities of the brain. Studies have shown that the psychological content expressed by explicit behaviour such as language is only a very small part of human psychology, while unconscious or subconscious activities account for more than 90% of human consciousness activities. Existing communication research can only obtain data through external observation or questionnaire survey. This kind of research is often limited to the evaluation of the psychological effects that people realize, and it is impossible to accurately explore the subconscious model of the effect of communication and its effects. Brain activity is still a “black box” for communication researchers.

**Figure 1:**
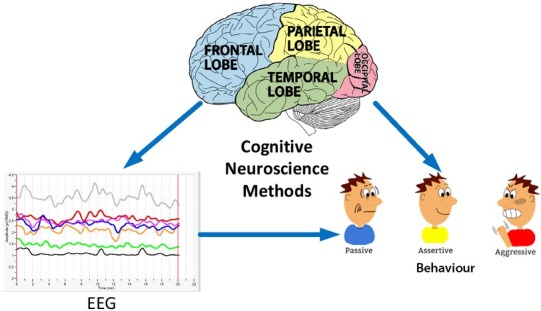
Cognitive Neuroscience Methods

The goal of cognitive neuroscience is to open the black box of human brain thinking and explore the material basis of human cognitive activities. It has two direct sources of discipline, cognitive neuropsychology and neuroscience. One of the challenges faced by human science is to understand the relationship between consciousness and the brain [9, 10]. From ancient Greece and China’s pre-Qin period, there was preliminary thinking on this issue. However, the scientific exploration of the relationship between the mind and brain began with the study of different cognitive impairments caused by the damage to different parts of the brain in the 19th century. The method of cognitive neuroscience is shown in Figure 1.

However, exploring the relationship between the mind and the brain from the scientific point of view began in the 19th century, and the damage to different parts of the brain led to the study of different cognitive function defects. In the middle of the 20th century, cognitive science began to systematically study the perception, attention, memory, movement, language, thinking, decision-making, motivation, emotional processes and structure of humans and animals. Based on these issues, psychology and language were assembled. Researchers in academia, anthropology, computer science, neuroscience, and other basic sciences have achieved a cross-disciplinary integration and integration in the history of science.

Mainstream journalism and communication studies mainly focus on the conscious content of communication. Recent studies have shown that beyond the explicit dimension of communication, the subconscious emotional dimension often plays a very important role. This dimension is often difficult to observe and capture by researchers. For example, in advertising, there is a rational dimension of product information dissemination, there is an emotion of brand image communication, and there is also an unconscious dimension, which can neither be attributed to rationality nor difficult to be attributed to emotion, but often becomes the real driving force for purchase, and this is precisely It is the important purpose of commercial advertising.

Many scholars and people in the industry have intuitively realized the existence of the third dimension. They know from experience and intuition that those seemingly accidental and irrelevant factors often play a vital role in the effectiveness of advertising, but they cannot be grasped by traditional research methods. Live them. The reason is that neurophysiological processes cannot be described by the coding of questionnaires or interviews, and unconscious purchase motives are more difficult to capture by means of observation or introspection. Observing the subconscious mind by means of consciousness seems to be a paradox in itself. This is a bottleneck problem that has long influenced the humanities and social sciences to study human cognition.

Through the measurement of computer, eye movement or galvanic skin, the subconscious mind of the subject is described, and this “third dimension” is captured. Such research does not rely on the conscious response of the subject, relying on what psychological feelings and psychophysiological reactions they claim to have, but rather obtaining continuous data of physiological tests without interference from the process of watching TV commercials.

**Figure 2:**
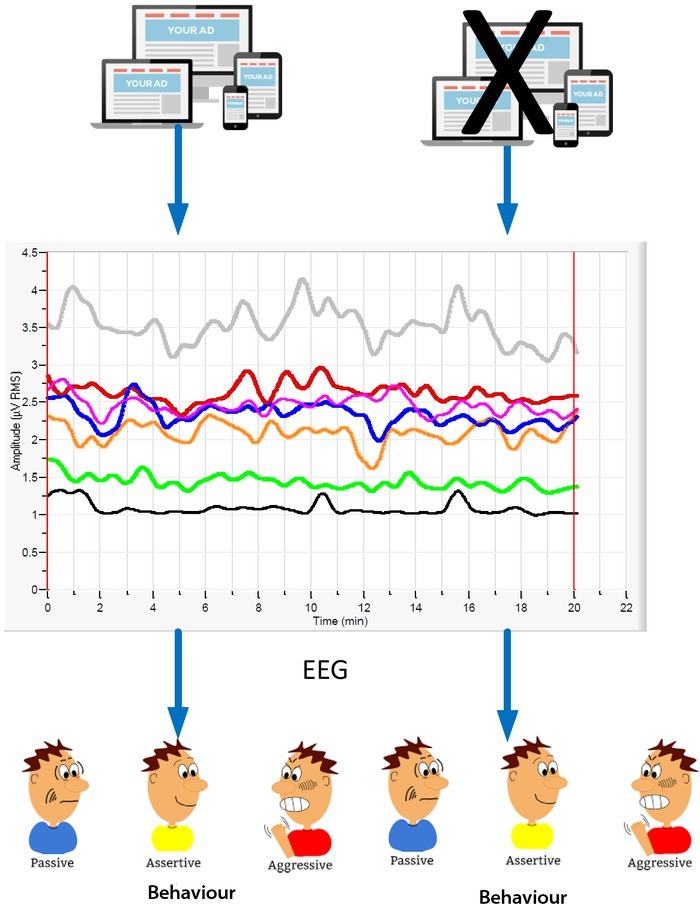
The emotional response of the subjects to the news

The mainstream of journalism and communication research focuses on the conscious content of communication, such as information and expressed emotions. Recent studies have shown that the subconscious emotional dimension or reflection without awareness often plays an important role in addition to the conscious dimensions of the communication [11]. This dimension is often difficult to observe and capture by researcher, but it can be investigated due to cross-disciplinary application of technologies including communication, cognitive neuroscience, and computer science. The electroencephalogram (EEG) test is the application of the difference between the left and right cerebral cortical potentials on the subject’s emotional approach and avoidance principle, recording the positive and negative effect of subjects’ emotion. The skin conductance test reflects the emotional evocativeness of subjects when viewing different advertising content. The experimental results are shown in Figure 2.

The first row of data in Figure 1 shows that the advertisement starts with a street scene shot with guitar accompaniment, which evokes a strong positive emotion. This shows that the effectiveness of advertising has far exceeded the conscious emotional design expectation. Further comparative experiment also shows that if the illogical symbol of the frog in the advertisement is deleted, the positive emotions of the audience on the product will decrease significantly [12].

## 3 Cognitive Neuroscience Method in Computational Communication Research

### 3.1 Cognitive neuroscience

**Figure 3:**
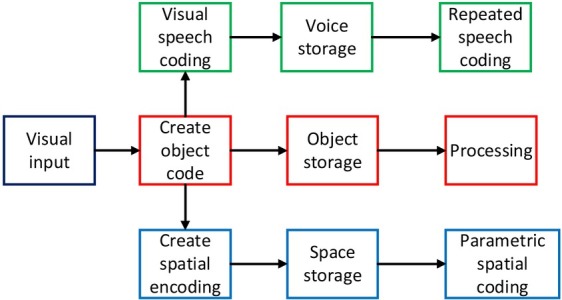
Working principles of cognitive neuroscience

In the last decades of the 20th century, Cognitive neuroscience has ready been applied to communication research on measuring short-term and long-term effects in the labs. New technologies of cognitive neuroscience in the 21st century can not only describe the subconscious mind of communication more accurately in the researches on advertisements, websites, and media users, but also measure the effect of communication more

objectively. This opens up a new paradigm for computational communication research based on laboratory data validation, which can be applied more widely in communication out of labs. The value of the paradigm lies in sharing the strengths of big data technology and cognitive neuroscience, that is, building up the powerful data base of media users and utilizing it to support cognitive neuroscience research on communication behaviours and effects. The exploration of introducing the experimental method of cognitive neuroscience into communication has achieved some encouraging results. And some research conclusions may even impact communication science disruptively. The realization process of cognitive neuroscience is shown in Figure 3.

### 3.2 Application of cognitive neuroscience in improving the content of communication

**Figure 4:**
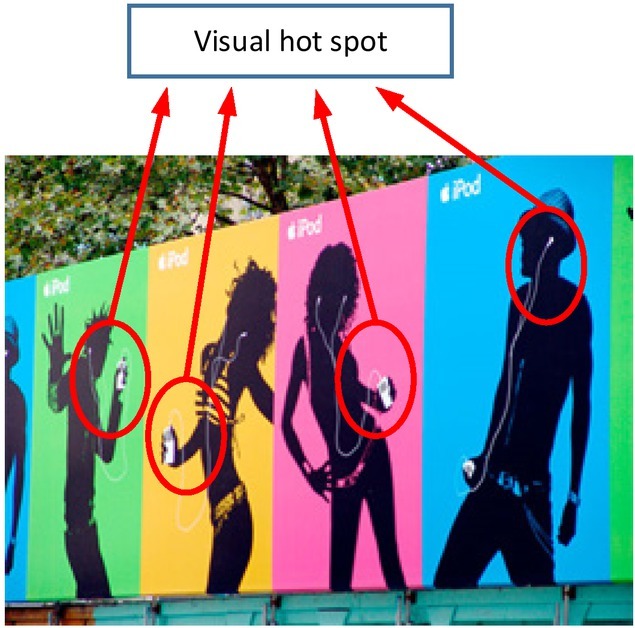
Eye movement experiment of beauty model plane advertisement

The results of eye movement experiment on print advertisements show that if the additional supporting graphics of ads (such as advertising models and colours) are excessively outstanding, they may significantly reduce the attentions on advertisers’ products and brand logos, though they could make advertisements more attractive. Thereby beautiful ad contents may fail to have the expected effectiveness. The eye-movement experiment on the contents of communication aims at obtaining audiences’ instant visual reactions to print ads. It can provide objective and accurate heat map presentation and data processing of information content in different regions. Specifically, how much attention is paid to different regions of interest can be presented. Based on the experiment, researchers and advertisers can find the interference regions in the ad contents and improve the design of advertising. The eye tracking results for print advertisements are shown in Figure 4.

### 3.3 Application of cognitive neuroscience in studying web users

**Figure 5:**
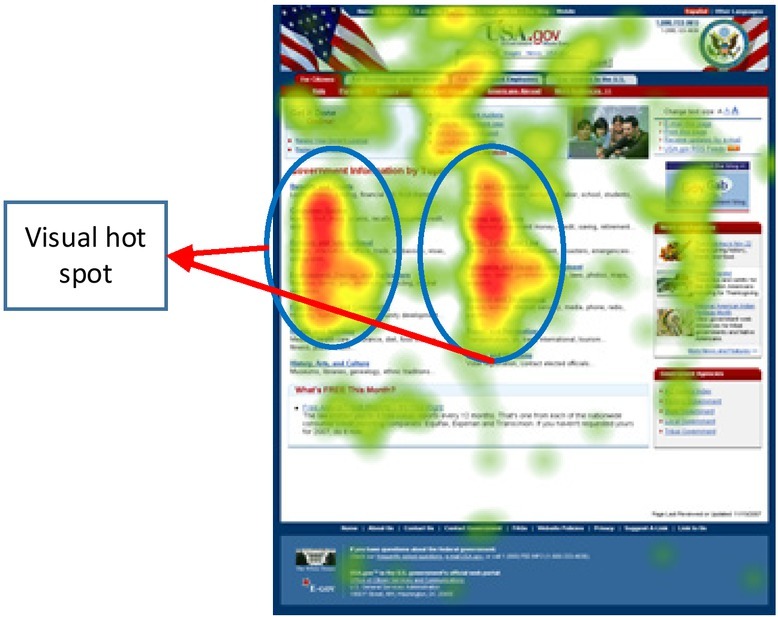
Eye movement tracing heat map

The research result of the “F-curve” of the web page layout can exert disruptive impact on the idea that the top-left corner is the first focus of reading. Traditionally, the top-left propriety or the Z-shaped trajectory of audiences’ text-reading

line is the news editors’ experience and practice in the print media era, and also the routine in the instructions of journalism and communication. However, in the network context this reading habit is just a hypothesis that has not been experimentally confirmed. Web users create another reading track in the experiment based on cyber data statistics and eye tracker. The research shows that similar “F” shaped line of sight has a high probability of reappearance. Similar researches have a certain guiding role in the arrangement of web pages and the layout of newspaper. The eye tracking results for web journalism is shown in Figure 5.

### 3.4 Application of cognitive neuroscience in investigation of the effect of communication

**Figure 6:**
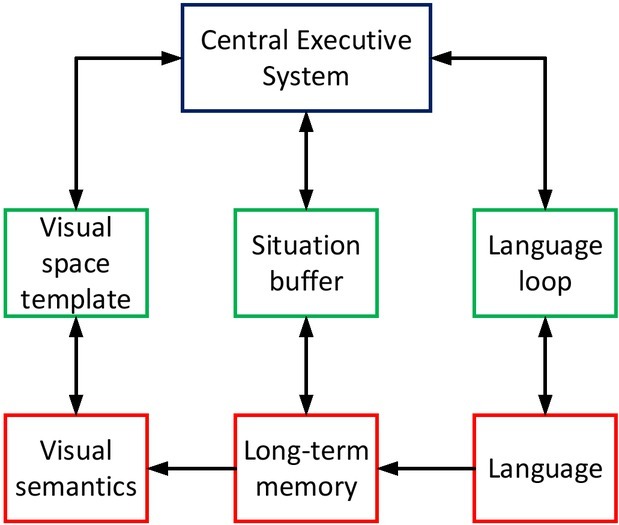
Application of four element model of cognitive neuroscience in data news analysis

How to effectively predict the effects of communication has always been a concern in the media corporations and academia. For example, the forecasting of the audience rating effect is a focus for producers, broadcasters, advertisers and viewers. Researchers can measure and collect audiences’ neurological responses to online and offline contents by measuring the EEG, skin conductance and eye movement. And then they apply calculation method of data for processing, analysing and visualizing the neurological response results, so as to provide reference for the video and news platform to predict the effects. An application example of the quadruple model of cognitive neuroscience is shown in Figure 6.

The experiments of a research team of Zhejiang University in China have found that the eye movement physiological test data of small-scale sample can reflect the audience rating of large-scale sample to a certain extent [13]. First of all, the index data of the number of gazing points is consistent with the order of the change in audience rating and these two have shown significant correlation. Secondly, the number of gazing points has a significant difference among different television

shows, making it easy to distinguish viewers’ preference to different television shows. Thirdly, the significant correlation between the time index before the first gaze, the audience rating and the number of gazing points also confirms the validity of the eye movement experiment method for predicting the audience rating. The research also finds that high investment and story popularity do not necessarily lead to high audience rating.

## 4 Example Analysis

In order to verify the method in this paper, the form of questionnaire will be used to analyse and the specific survey results are shown in Table 1.

**Table 1:** Survey results

Content	Totally agree	More agree	General	Disagre	strongly disagree
Like the Web News	26	23	13	2	1
You are easy to understand the news	23	24	14	6	1
More concerned about the news are very good and they have good design	11	16	19	9	2
your focus is more focused with the object	12	18	26	8	3
The use of cognitive neuroscience makes it easier for you to understand the meaning of the news	18	21	24	15	4
After using the cognitive neuroscience, your understanding of the content is easier	20	26	13	3	1
The use of cognitive neuroscience makes you more memorable to the content	16	17	18	4	1

By observing the above survey statistics, the following conclusions can be obtained: the application of cognitive neuroscience to the research on data journalism can enable

people to better understand the meaning of journalism. In addition, the changes in the level of people’s understanding of journalism with the increase of time are also studied and the specific results are shown in Table 2.

**Table 2:** The change in the degree of people’s understanding of the news with time

Parameter				Time (month)		
	1	2	3	4	5	6
Level 1	70	80	90	92	100	100
Level 2	65	85	91	93	95	96
Level 3	30	50	60	79	80	85
Level 4	98	93	98	99	100	100

**Figure 7:**
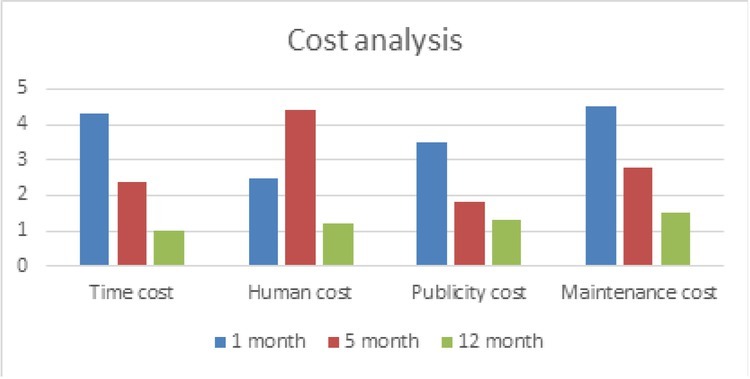
Cost analysis of the new paradigm of data journalism based on cognitive neuroscience

From the above table, it can be seen that as time increases, people’s understanding of level 1 and level 2 will continue to increase and the highest accuracy can reach 100%. This is because cognitive neuroscience can better study these two levels of understanding. Through continuous enhanced learning, people will gradually understand the meaning behind the journalism. Finally, the cost analysis is conducted on the new research paradigm of computational communication as well as data journalism based on cognitive neuroscience designed in this paper and the specific analysis results are shown in Figure 7.

It can be seen from the above analysis that the longer it takes to use the method proposed in this paper, the lower the overall cost will be. This is because the skilled mastering of the method proposed in this paper can effectively reduce time, labour, operating and maintenance costs. Therefore, the method in this paper can be effectively promoted, and with the increase of the promotion time, the benefits obtained from the promotion will also continue to increase.

## 5 Conclusions

The introduction of cognitive neuroscience will be beneficial to the exploration of the underlying mechanism of interdisciplinary dialogue and communication. However, it is obvious that to complete such a major theoretical adjustment and construction, it is obviously not enough to rely on the ideas and methods of cognitive neuroscience. On the one hand, any research method has its limitations. Cognitive neuroscience can’t arrange journalism communication to solve all problems. On the other hand, cognitive neuroscience is still in the exploratory stage, and relevant theories and methods need to be further improved. The important issues of the series have yet to be further explored. We draw on cognitive neuroscience, mainly to provide a new theoretical perspective and research method for the audience research, media research and effect research of the existing news communication, with the help of its “intuitive human brain” method, so as to try to spread the news. The basic paradigm of learning and practical application of learning establishes a new paradigm.

The validity of the new paradigm depends not only on the cognitive neuroscience itself, but also on the rational framework of basic theories of communication. According to the basic problem orientation and the discipline nature of computational communication science, the combination of cognitive neuroscience and communication symbols is a direction that is expected to achieve major breakthroughs. Without symbols, there will be no communication and human society; without communication, the symbols will lose their meaning. Therefore, while continuing to promote the study of communication semiotics and using it as the basic framework for the basic theory construction of communication science, we should also draw lessons from cognitive neuroscience and other disciplines to conduct more objective studies and detailed description on symbol content, meaning and communication effect, promoting the study of semiotics to a new stage based on experimental data.

## References

[1] Leu D. J., Forzani E., Rhoads C., Maykel C., Kennedy C., Timbrell N. (2015). The new literacies of online research and comprehension: Rethinking the reading achievement gap. Reading Research Quarterly.

[2] Spunt R. P., Adolphs R. (2017). A new look at domain specificity: insights from social neuroscience. Nature Reviews Neuroscience.

[3] Gershman S. J., Horvitz E. J., Tenenbaum J. B. (2015). Computational rationality: A converging paradigm for intelligence in brains, minds, and machines. Science.

[4] Du X., Zhu Y., Peng Z., Cui Y., Zhang Q., Shi Z., Guan Y., Sha X., Shen T., Yang Y., Li X., Wang Z., Li X., Liu G. (2018). High concentrations of fatty acids and beta-hydroxybutyrate impair the growth hormone-mediated hepatic JAK2-STAT5 pathway in clinically ketotic cows. Journal of Dairy Science.

[5] Collins A. G., Frank M. J. (2018). Within-and across-trial dynamics of human EEG reveal cooperative interplay between reinforcement learning and working memory. Proceedings of the National Academy of Sciences.

[6] Fitch W. T. (2014). Toward a computational framework for cognitive biology: unifying approaches from cognitive neuroscience and comparative cognition. Physics of life reviews.

[7] Johnson M. (2014). The forest for the trees. comment on “toward a computational framework for cognitive biology: unifying approaches from cognitive neuroscience and comparative cognition” by w.t. fitch. Physics of Life Reviews.

[8] Panksepp J. (2015). Toward the constitution of emotional feelings: Synergistic lessons from Izard’ s differential emotions theory and affective neuroscience. Emotion Review.

[9] Koller M., Walla P. (2015). Towards alternative ways to measure attitudes related to consumption: Introducing startle reflex modulation. Journal of agricultural & food industrial organization.

[10] Ohme R. (2009). The Unconscious as the Third Dimension in Advertising. American Academy of Averting AAA Newsletter.

[11] Josée Leclerc. (2006). The unconscious as paradox: impact on the epistemological stance of the art psychotherapist. Arts in Psychotherapy.

[12] VanCleave D. S. (2016). Contributions of Neuroscience to a New Empathy Epistemology: Implications for Developmental Training. Advances in Social Work.

[13] Li S. (2016). Cognitive Neuroscience and New Paradigm in Journalism and Communication. Journalism and Writing.

